# A Case of Legionella Pneumonia in an Older Patient Without Typical Exposure to a Susceptive Environment

**DOI:** 10.7759/cureus.27541

**Published:** 2022-07-31

**Authors:** Yasuhisa Nakano, Kota Saka, Fumiko Yamane, Chiaki Sano, Ryuichi Ohta

**Affiliations:** 1 Medicine, Shimane University Faculty of Medicine, Izumo, JPN; 2 Family Medicine, Shimane University Faculty of Medicine, Izumo, JPN; 3 Community Care, Unnan City Hospital, Unnan, JPN; 4 Community Medicine Management, Shimane University Faculty of Medicine, Izumo, JPN

**Keywords:** family medicine residency, general physician, rural hospital, exposures to susceptive environment, sewage and soil use, older patient, legionella pneumonia

## Abstract

Legionella pneumonia is a potentially fatal form of pneumonia that causes various clinical symptoms and is often difficult to diagnose. For the diagnosis, it is important to inquire about the patient's history of exposure to sewage or soil, although there are rare cases of Legionella pneumonia with no history of exposure. In this study, we present a case of Legionella pneumonia in a 72-year-old man with no history of wastewater exposure from public baths or other sources. The patient presented to our emergency department with fever, chills, and shivering. The antigen test of the urine for Legionella was negative, and chest radiography showed patchy infiltrates in the right lower lung field that was suspicious for pneumonia. The patient was treated with intravenous ceftriaxone (2 g/day) for right-sided pneumonia and was intubated on day 1 due to poor oxygenation and a tendency towards exacerbation to acute respiratory distress syndrome. The fever resolved after day 3 (36.4-36.9°C), and the patient was extubated on day 6. A positive sputum polymerase chain reaction (PCR) test for Legionella deoxyribonucleic acid (DNA) (type 1) was performed on day 6, and levofloxacin and dexamethasone therapy was administered. After completing a 10-day course of levofloxacin, the patient's symptoms were cured. Although it is important to note the patient’s background, symptoms, and information on the clinical course, including laboratory values, to include a diagnosis of Legionella pneumonia, it is impractical to suspect Legionella pneumonia in all patients admitted to the hospital with pneumonia and to administer new quinolone antimicrobials. However, it is important to re-evaluate the diagnosis and intervene in treatment when β-lactam antimicrobials are ineffective or when extrapulmonary symptoms are present, as in this case.

## Introduction

*Legionella pneumophila* causes a fatal form of pneumonia that presents with a wide variety of clinical symptoms and is often difficult to diagnose. The main symptoms are similar to those of common variants of pneumonia, including cough and fever, while extrapulmonary symptoms such as gastrointestinal symptoms (diarrhea and abdominal pain) and neurological symptoms (headache and disorientation) are also relatively common [1-3].

Legionella grows within phagocytic cells such as alveolar macrophages. Thus, the effect of β-lactams, and aminoglycosides, which have low intracellular activity, is reduced. Therefore, the Infectious Diseases Society of America recommends fluoroquinolones or macrolides as the first-line treatment for *Legionella pneumophila* [4].

A history of the source of infection is important for diagnosing pneumonia caused by* Legionella pneumophila*, which, along with most Legionella species, are often found in water bodies, mainly lakes, streams, and artificial reservoirs. Legionella can live planktonically or as an intracellular parasite within protozoa [5], and their growth is strongly enhanced in warm temperatures (25-42°C) and stagnant water [6].

Therefore, obtaining a patient history of sewage and soil exposure is important, although rare cases of *Legionella pneumophila* have been reported with no history of exposure. This study presents a case of Legionella pneumonia in a patient with no history of sewage exposure from a public bathhouse or other sources.

## Case presentation

A 72-year-old man presented to our emergency department (ED) with a chief complaint of fever. He was diagnosed with a mild common cold, fever, and dehydration and was prescribed acetaminophen for symptom relief. The patient rested at home, but during the night on the same day, tremors appeared, and the family called for emergency medical assistance. The patient had a carotid artery stenting (CAS) history and a cerebral ischemic attack after CAS (X-7 years). He was started on three antiplatelet agents at that time, and in-stent plaque disappearance was confirmed. A colon polypectomy (X-6 years) was performed. His medications included atorvastatin (10 mg), rebamipide (100 mg), magnesium oxide (330 mg), and amlodipine besylate (2.5 mg). The patient was COVID-19 antigen-negative and reported smoking five cigarettes per day and occasional alcohol consumption.

On arrival at the ED, vital signs were as follows: temperature was 40.5°C, pulse was 94 bpm, blood pressure was 123/57 mmHg, respiratory rate was 26 times/min, SpO_2_ was 90% on room air, and he was fully conscious. Physical examination revealed chills, shivering, and drooping of the right mouth. There was no cough, and the pharynx was mildly hyperaemic and painless. Abdominal pain was also noted. Lung sounds were inaudible throughout the chest. Aspiration was not observed. No obvious insect bite was observed. Initial blood findings included an elevated white blood cell count and C-reactive protein (CRP). Other findings of platelet count and serum sodium were decreased, BUN and creatinine were elevated, and urinary Legionella antigen test was negative (Table 1).

**Table 1:** Patient's initial laboratory data RDW: red cell distribution width; CRP: C-reactive protein

Laboratory parameter	Level	Reference ranges for blood tests
White blood cell (x10^3^/μ)	14	3.5-9.8
Red blood cell (x10^6^/μ)	4	4.10-5.30
Hemoglobin (g/dL)	12.8	13.5-17.6
Hematocrit (%)	38	36-48
Mean corpuscular volume (fL)	95	82-101
RDW (%)	14.5	11.5-14.5
Platelet (x10^4^/μ)	25.2	13.0-36.9
Total bilirubin(mg/dL)	1	0.2-1.2
Aspartate aminotransferase (IU/L)	22	8-38
Alanine aminotransferase (IU/L)	14	4-44
Alkaline phosphatase (U/L)	70	38-113
Gamma-glutamyl transpeptidase (IU/L)	28	16-73
Lactate dehydrogenase (U/L)	168	106-211
Total protein (g/dL)	6.7	6.6-8.1
Albumin (g/dL)	3.7	3.9-4.9
Blood urea nitrogen (mg/dL)	18.9	8.0-20.0
Creatinine (mg/dL)	1.02	0.40-1.10
Serum Na (mEq/L)	134	135-147
Serum K (mEq/L)	3.8	3.3-4.8
Serum Cl (mEq/L)	99	98-108
Serum Ca (mg/dL)	9.2	8.8-10.2
CK (U/L)	84	56-244
CRP (mg/dL)	11.59	≥0.3
Estimated glomerular filtration rate (mL/min/1.73 m^2^)	55.6	≤60

**Figure 1:**
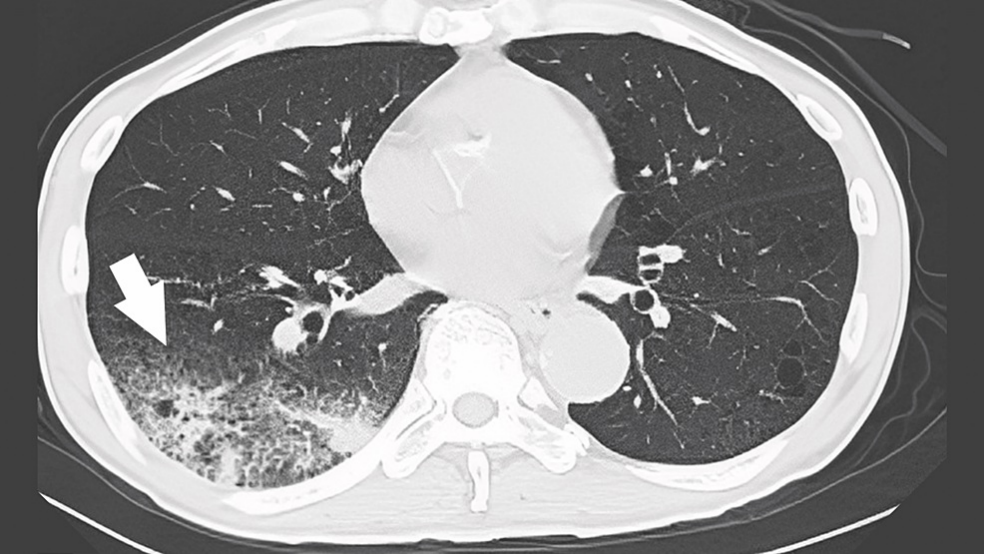
Results of chest computed tomography (day 1) showing right lower lobe infiltration

Chest radiography suggested pneumonia in the right lower lung field, and chest computed tomography (CT) revealed an extensive infiltrative shadow in the right lower lung field and surrounding frosted shadows (Figure 1). The patient was treated with intravenous ceftriaxone (2 g) for pneumonia in the right lung.

On admission (day 0), respiratory sounds in both lung bases decreased, coarse crackles were heard in the right lung, wheeze was not heard, and heart sounds were only systolic ejection murmurs without radiation to the necks.

**Figure 2:**
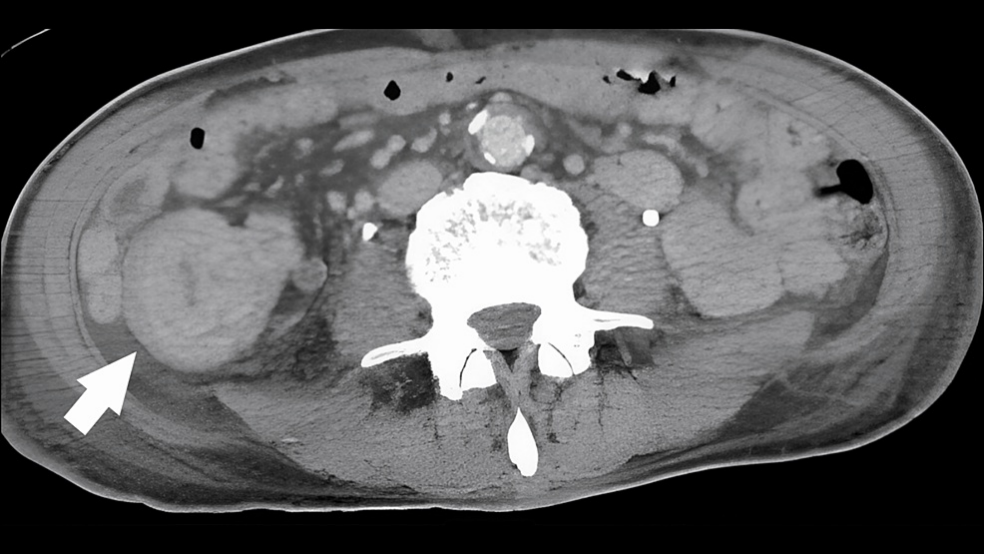
Abdominal computed tomography showing a mass in the right lower abdomen

On day 1, SpO_2_ dropped to the 70-80% range with 5 L of oxygen; the volume was increased to a 10 L reservoir without improvement in oxygenation. The patient was intubated because of poor control of pneumonia, poor oxygenation, and a tendency toward exacerbation. Physical examination revealed that the patient again developed chills and shivers and became agitated. There was no difference in bilateral lung sounds. No obvious pneumothorax was observed. Right crackles without jaundice were also observed. The additional sputum examination results were negative for tuberculosis. Blood, urine, and sputum smears revealed no significant bacteria. Because of the dry cough symptoms and relatively slow pulse rate, atypical pneumonia was considered, and levofloxacin 500 mg was administered. Because the patient had a cat, we selected an antibacterial agent covering Q fever and Pasteurella. No wounds were observed, but 100 mg of minocycline was added to cover rickettsia. Cefmetazole (4 g) was added for possible bacterial translocation by the enterobacteria. Dexamethasone (33 mg) was added for acute exacerbation of acute respiratory distress syndrome (ARDS) and chronic obstructive pulmonary disease (COPD). CT, which was performed because of abdominal pain, showed a mass lesion in the ascending colon with surrounding edematous changes and lymphadenopathy (Figure 2).

**Figure 3:**
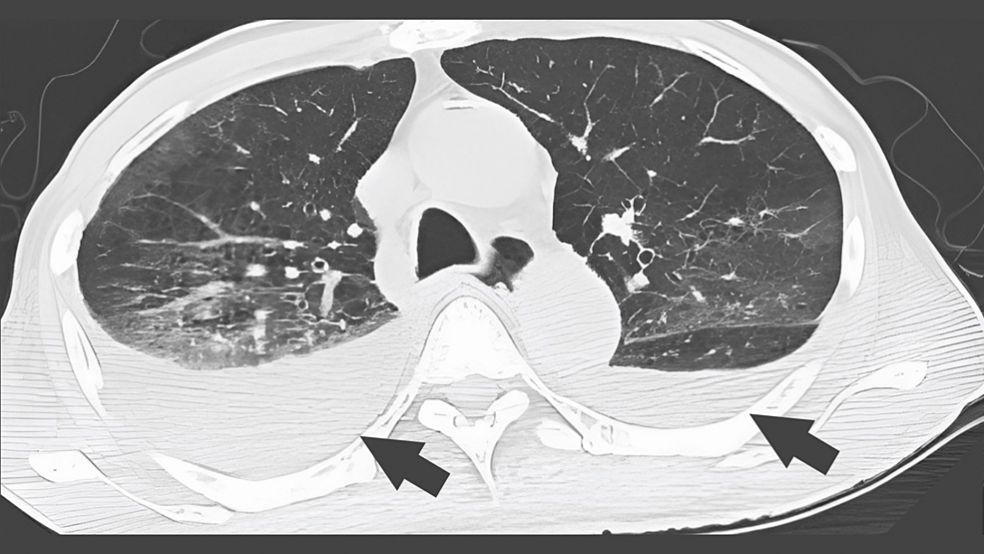
Chest computed tomography showing the bilateral lung frosted shadows and pleural effusions.

The fever resolved after day 3 (36.4-36.9°C), respiratory status started at FiO_2_ of 0.4, and the patient was extubated on day 6. On day 6, the sputum PCR test was positive for Legionella DNA (type 1), minocycline was discontinued, and the patient was treated with levofloxacin and dexamethasone. A CT image of the chest on day 9 showed the bilateral lung frosted shadows and pleural effusions (Figure 3).

After completing a 10-day course of levofloxacin, the patient was stable and was transferred to the surgery department for the preparation of the investigation of a mass lesion in the ascending colon on day 16 of admission.

## Discussion

This patient was one of the most difficult to diagnose cases of Legionella pneumonia because of the variety of clinical symptoms associated with Legionnaires' disease, including fever, shivering chills, abdominal pain, and hyponatremia, as well as the patient's lack of history of using public baths or well water.

Legionella infection is associated with two major factors: (i) the patient's exposure history and (ii) the patient's immune status [7]. Concerning the patient's exposure history, it is important to ask whether the patient had used public baths or had consumed well water. Factors compromising a patient's immune defense include age, smoking, chronic lung disease, cardiovascular disease, and renal disease [7]. In the present case, the patient was atypical with no history of using public baths and a negative urine Legionella antigen test; therefore, the general history may not apply. Initially, Legionella infection was considered negative, but because the patient was older and had a history of smoking and cerebrovascular disease, it was necessary to consider whether there was any atypical exposure history to be included in the differential diagnosis.

*Legionella pneumophila* can have a wide variety of clinical manifestations, and knowledge of the frequency of each clinical manifestation may be helpful for the diagnosis. The symptoms (frequency) of *Legionella pneumophila* reported in previous US studies included cough (67%), purulent sputum production (27%), pleurisy (21%), myalgia (51%), headache (43%), digestive symptoms (19%), chills (59%), and confusion (25%) [8,9]. Legionella pneumonia is also characterized by a lower incidence of purulent sputum (Legionella, 27%; pneumococcal pneumonia, 64%). Legionella pneumonia is associated with bradycardia, hypoxemia, and elevated liver enzyme levels [8]. However, many of these mechanisms are not clearly understood. For example, the syndrome of inappropriate antidiuretic hormone has been considered a factor in hypoxemia, but no conclusive reports indicate this, and some reports are negative [8]. Laboratory values also showed a mean leukemic cell count x10^9^ ± standard deviation (SD)/L (12.8 ± 6.7), creatinine concentration ± SD mmol/L (129.0 ± 106.9), Na concentration ± SD mmol/L (132.5 ± 4.2) and lactic acid concentration ± SD μkat/L (11.2 ± 4.8). It is important to monitor and follow the course of the disease, especially in Japan, as CRP and sodium are excellent predictors for the diagnosis of *Legionella pneumophila* [10].

The diagnostic and treatment strategies for *Legionella pneumophila* infection in remote hospitals are described below. The first step was to test for Legionella spp. Nucleic acid identification tests (loop-mediated isothermal amplification {LAMP} and PCR methods) using urine and sputum specimens can detect *Legionella pneumophila *types other than serogroup 1 and Legionella species other than *Legionella pneumophila*. In Japan, the LAMP method was covered by insurance in 2011, and some regional health laboratories have used either PCR or LAMP methods. In Japan, a test kit is commonly used to detect Legionella antigens in urine; however, it is important to understand the characteristics of the test kit. The urinary antigen kit used in this study is an immunochromatographic urinary antigen test. It only tests for serogroup 1 (serotype 1) of *Legionella pneumophila*. As serotype 1 is the cause of approximately half of the *Legionella pneumophila* cases in Japan, it is possible that some types of Legionella could be judged as negative. A urine antigen test capable of detecting 15 serotypes (1-15) was launched in Japan in 2019 [10]. The characteristics of the test kits used in each facility were assessed. However, most hospitals in remote areas have not introduced this method because of a combination of costs. Therefore, diagnosing patients using limited resources such as urinary antigen kits is necessary.

Next, the medical history of the patients is discussed. Patients in remote hospitals are often older people, and in many cases, obtaining their exposure history is impossible. Therefore, it is necessary to follow the clinical course of *Legionella pneumophila* carefully, including low Na + and high CRP levels, which are characteristic of *Legionella pneumophila*. Atypical pneumonia may be aggressive in the older population. Finally, the treatment options are presented. Appropriate antimicrobial treatment for community-acquired pneumonia in normal adults improves the patient's clinical course (especially vital signs, cough symptoms, and chest radiographs) within 48-72 h [11,12]. Therefore, patients who do not show any clinical improvement within 72 h of antimicrobial treatment should be considered non-responders, and treatment should be initiated with atypical pneumonia in mind. It is inappropriate to suspect *Legionella pneumophila* in all patients admitted to the hospital with pneumonia and administer new quinolone antimicrobials. It is important to develop a treatment strategy for atypical pneumonia when β-lactam antimicrobials are ineffective or when extrapulmonary symptoms are present, as in the present case. The appropriate antibiotic coverage should be considered because testing for all *Legionella pneumophila* serogroups is not readily available and straightforward in rural contexts.

Furthermore, older patients’ symptoms could differ from those of younger patients, which should be conveyed to medical professionals and lay people. Symptom detection is essential for the effective diagnosis of atypical pneumonia, including Legionella pneumonia. Older patients tend to have vague symptoms of various diseases and use self-management [13], especially in rural contexts [14]. Medical and public health professionals should provide appropriate information regarding effective help-seeking behaviors for vague symptoms and improve the care of older patients with atypical pneumonia [15].

## Conclusions

The diagnosis of Legionella pneumonia is complicated by the variety of clinical symptoms associated with Legionnaires' disease, including fever, shivering chills, abdominal pain, and hyponatremia, as well as patient backgrounds, such as the patient's exposure history, aging, and the other patient susceptibility to infection. It is important to pay close attention to the patient's symptoms and clinical course, including the laboratory results. However, it is not realistic to suspect Legionella pneumonia in all patients admitted to the hospital with pneumonia and to administer new quinolone antimicrobials. It is important to re-evaluate the diagnosis and intervention in the treatment of cases in which β-lactam antimicrobials are ineffective, or extrapulmonary symptoms are present, as in the present case.
